# Splenic extramedullary hematopoiesis in myocardial infarction

**DOI:** 10.1016/j.fmre.2025.07.006

**Published:** 2025-07-22

**Authors:** Nan Wang, Alan R. Tall

**Affiliations:** Division of Molecular Medicine, Department of Medicine, Columbia University Irving Medical Center, New York, NY 10032, USA

Myocardial infarction (MI) as a result of atherosclerotic plaque rupture or erosion is the major cause of mortality and morbidity in cardiovascular disease. MI-induced ischemia and progressive myocardial cell death rapidly trigger a series of immune responses, including increased production and release of inflammatory mediators, endothelial cell activation, and increased recruitment of myeloid cells from circulation [1]. The recruitment of circulating myeloid cells into the heart, primarily to the infarcted area, is associated with a mobilization of hematopoietic stem and progenitor cells (HSPC) and release of mature myeloid cells from bone marrow and spleen. The early phase of MI is dominated by the recruitment of inflammatory neutrophils and monocytes/macrophages with high proteolytic and phagocytic activity, facilitating the clearance of damaged tissue and dead cells. This initial inflammatory clean-up phase will gradually transition into a reparative healing phase with inflammation resolution and tissue repair. While the initial recruitment of circulating myeloid cells is associated primarily with the release of myeloid cells from bone marrow and spleen, continued recruitment requires enhanced hematopoiesis and myeloid cell production. A major source for this is increased extramedullary hematopoiesis (EMH) in the spleen. Mobilization and release of HSPCs from bone marrow into circulation during the early phase of MI seed the spleen and facilitate splenic EMH. In response to MI, the spleen overall contributes as much as 50% of all myeloid cells that enter the ischemic myocardium [1]. Splenic EMH is required for myocardial infarct healing, since abrogation of splenic EMH has proved deleterious for infarct healing and accelerated the evolution of heart failure [2].

Due to its functional significance in health and disease, hematopoiesis has been extensively investigated at steady state or under stress, but the focus has been primarily on hematopoiesis in bone marrow. After MI, dead and dying cells in the infarcted myocardium and recruited inflammatory myeloid cells release various inflammatory mediators, including cytokines, chemokines, and damage-associated molecular patterns, which act on their respective receptors on hematopoietic and niche cells in the bone marrow to promote hematopoiesis and leukocyte release. MI also increases the sympathetic nervous system activity and attenuates the production of the HSPC retention factor CXCL12 from niche cells, leading to increased mobilization of HSPCs into circulation. In contrast, there is limited understanding of how EMH at steady state or under stress is regulated. In general, EMH in the spleen at steady state is considered to have a minor role in hematopoietic homeostasis relative to hematopoiesis in the bone marrow. However, EMH in the spleen plays a dominant role under hematopoietic stress, particularly when an acute and emergent increase in hematopoiesis and myeloid cell production is required, such as in blood loss or anemia, infection, myeloablation, and MI [1]. HSPCs mobilized from the bone marrow and seeded in the spleen require stem cell factor (SCF) from endothelial cells and *Tcf21*^+^ stromal cells and CXCL12 from *Tcf21*^+^ stromal cells around sinusoids in the red pulp to undergo EMH [3]. Endothelial cells and *Tcf21*^+^ stromal cells thus create a perisinusoidal EMH niche in the spleen. Conditional deletion of *Scf* from spleen endothelial cells, or of *Scf* or *Cxcl12* from *Tcf21*+ stromal cells, severely reduced splenic EMH and reduced blood cell counts without affecting bone marrow hematopoiesis [3]. Nevertheless, how splenic EMH is regulated and whether this regulation impacts infarct repair and cardiac function following MI is still poorly understood.

A recent study by Lv et al. [4] represents a timely and elegant approach to answer this important question. As reported previously, they confirmed a MI-induced acute but mild spleen shrinkage on day 1, parallel to the increased release of myeloid cells from the spleen [5], and subsequent splenic EMH and increased spleen weight and volume in mice, in association with increased number of HSPCs, myeloid progenitors and mature myeloid cells in the spleen. MI did not change the bone marrow cellularity and HSPC numbers. To determine the role of splenic EMH in the recruitment of cardiac myeloid cells after MI, Tcf21^CreER^/Scf^flox/flox^ mice were used for conditional deletion of *Scf* in splenic Tcf21^+^stromal cells. SCF deficiency selectively reduced spleen weight and volume and the number of spleen but not bone marrow HSPCs, in association with decreased M2-like but increased M1-like cardiac macrophages. To assess whether this change was caused by reduced splenic EMH, GFP^+^ hematopoietic stem cells (HSC) isolated from the spleen were adoptively transferred to Tcf21^CreER^/Scf^flox/flox^ or the control mice. Following MI, the GFP^+^ cells were detected in the infarcted heart and in the peripheral circulation. However, the number of GFP^+^ cells in the infarcted area was lower in Tcf21^CreER^/Scf^flox/flox^ relative to the control mice, supporting that SCF deficiency impairs the EMH, and subsequently leads to reduced recruitment of monocytes/macrophages into the infarcted hearts after MI. Next, cardiac function was assessed following MI in these mice to evaluate whether splenic EHM impacts cardiac repair and its function after MI. Mice with SCF deficiency displayed impaired cardiac function, in association with greater infarct size, suppressed angiogenesis, enhanced cardiac hypertrophy and worsened survival. To exclude the potential Tcf21^CreER^ leaky expression effects, the authors went further to study WT mice that underwent splenectomy followed by transplantation of splenic tissue from Scf^flox/flox^ control or Tcf21^CreER^/Scf^flox/flox^ mice. The cardiac functional and cell composition phenotypes and peripheral blood monocyte profiles in the transplanted Tcf21^CreER^/Scf^flox/flox^ mice largely replicated that in non-transplanted Tcf21^CreER^/Scf^flox/flox^ mice, confirming the functional selectivity and specificity of Tcf21^CreER^/Scf^flox/flox^ in the spleen in this model. To understand the mechanism underlying the regulation of splenic EMH in response to MI, a ScRNA-seq analysis was performed on spleen-derived HSPCs, and this analysis unraveled a suppressed type 1 interferon (IFN) pathway and reduced expression of type 1 IFN-stimulated genes, particularly in a subset of HSCs with high stemness. The crucial role of type 1 IFN in the regulation of the splenic EMH after MI was confirmed by a conditional deletion of *Ifnar1* in hematopoietic cells, which increased spleen weight and the number of HSPCs in the spleen but not in the bone marrow. However, the concentration of type 1 IFNs in the spleen after MI did not show a significant change. Using sophisticated tandem liquid chromatography mass spectrometry, the authors identified prostaglandin I2 (PGI2) as the major decreased lipid metabolite in the spleen in response to MI. The following mechanistic studies suggested that MI decreases PGI2 generation. PGI2 acts on its receptor in HPSCs, activating transcription factor SP1 and promoting its target gene expression including *Stat1*. Cicaprost, a PGI2 receptor agonist, reversed the reduction of type 1 IFN target gene expression and, importantly, hematopoietic deletion of IP, the PGI2 receptor, resulted in increased splenic EMH, increased cardiac M2-like macrophages and decreased M1-like macrophages after MI, in association with reduced cardiac infarct size and improved cardiac function. Using genetic mouse models, this study clearly demonstrates that the lack of splenic EMH exacerbates myocardial injury, impairs cardiac repair and cardiac function after MI. The study also suggests that antagonism of hematopoietic PGI2 signaling may improve cardiac outcome after MI. However, it should also be recalled that inhibition of PGI2 production was thought to be responsible for the increased risk of MI and thrombosis associated with the use of commonly used COX2 inhibitors [6]. Thus, a deeper understanding of the sources and effects of PGI2 production in the spleen may be needed prior to further therapeutic development

This study provides important mechanistic insights into the role and regulation of splenic EMH in the post-MI cardiac recovery. Previous studies using splenectomy suggest a critical role of splenic EMH in post-MI cardiac repair and functional recovery [2]. Using genetic models and spleen tissue transplantation, this study unequivocally demonstrates the dominant role of splenic EMH in post-MI cardiac recovery. This study also raises interesting questions. While the recruitment of leukocytes following MI helps the clearance of dead cells and repair of damaged tissues, the immune cells show high diversity in cell identity and function. scRNA-seq analyses have identified up to 7 transcriptionally distinct subsets of monocytes/macrophages in the infarcted heart following MI in mice. Among these, a subset of interferon-inducible macrophages termed IFNIGs promotes inflammatory responses and exacerbates post-MI recovery [7]. The functional distinctiveness of the other 6 subsets remains to be investigated. The functional diversity may not be fully appreciated by the simplified M1/M2 classification. For instance, it might be more informative if the identity of M2-like macrophages, which may improve cardiac repair and function as suggested by the current study, could be identified by single-cell analysis and targeted genetically in MI models. Increased infiltration of the inflammatory neutrophils and monocytes from the spleen into atherosclerotic arterial walls after MI promotes atherosclerosis [8], and possibly is responsible for the increased rate of recurrent cardiovascular events following the initial MI or stroke. Single-cell analysis of atherosclerotic lesional leukocytes using this genetic spleen EMH model may help identify the splenic EMH mobilized pro-atherogenic immune cell sub-populations responsible for the exacerbated atherosclerosis. Additional questions may include whether and how PGI2 antagonism, which improves cardiac outcome after MI, impacts MI-exacerbated atherosclerosis.

Most studies on EMH in cardiac repair and function after MI are performed using animal models. Clinical studies revealed that patients with acute MI have more circulating HSPCs in their blood [8,9], similar to the observations in mice post-MI. Splenic hematopoiesis in humans has been indirectly shown via increased ^18^F-FDG (fluorodeoxyglucose) uptake in PET imaging after acute MI [10], and splenic ^18^F-FDG PET/CT signal correlates with echocardiographic indices of diastolic dysfunction [11]. World War II veterans who underwent splenectomy had an excess mortality from ischemic heart disease [12]. The translatability of the findings from mice to humans on the role of splenic EMH in post-MI cardiac recovery needs to be assessed with additional and rigorous studies. Nevertheless, these elegant animal studies provide important scientific rationale and mechanistic pathways to further investigate the role of EMH in human cardiovascular disease, with potential high rewards in cardiovascular disease therapy (Fig. 1).

**Fig. 1:**
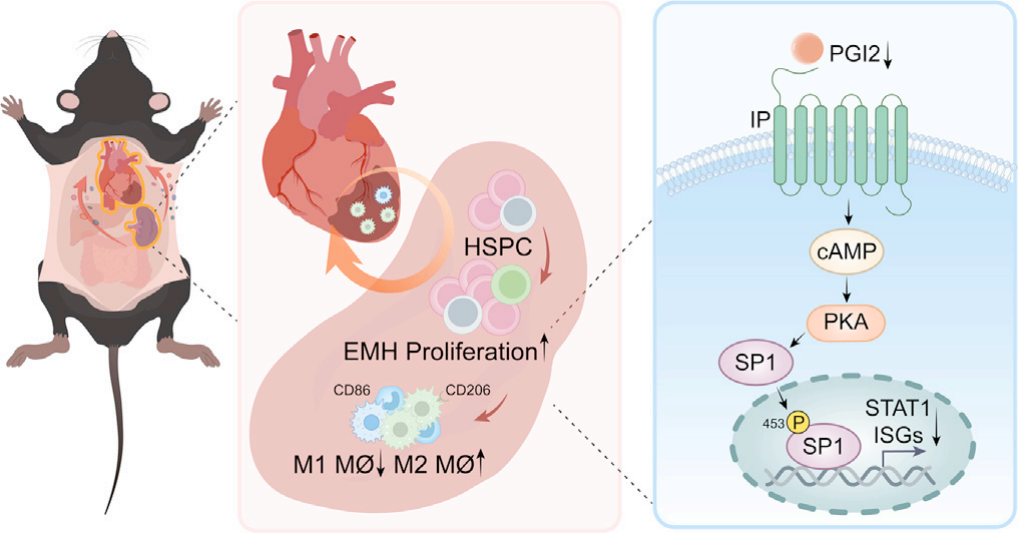
**Myocardial infarction (MI) mobilizes HSPCs from the bone marrow into circulation, which seed the spleen**. MI also leads to reduced PGI2 production and PGI2 signaling-promoted phosphorylation and activation of SP1. As a result, the expression of interferon-stimulated genes (ISGs), including STAT1, is reduced. Type 1 interferon signaling inhibits splenic EMH, and MI promotes splenic EMH by decreasing the expression of ISGs via reduced PGI2 production and signaling. Genetic disruption of splenic EMH decreases M2-like cardiac macrophages while increasing M1-like cardiac macrophages in the infarcted area following MI, exacerbating cardiac inflammation and impairing cardiac tissue repair and cardiac function.

## CRediT authorship contribution statement

**Nan Wang:** Writing – original draft. **Alan R. Tall:** Writing – original draft.

## References

[1] Poller W.C., Nahrendorf M., Swirski F.K. (2020). Hematopoiesis and cardiovascular disease. Circ. Res..

[2] Leuschner F., Rauch P.J, Ueno T. (2012). Rapid monocyte kinetics in acute myocardial infarction are sustained by extramedullary monocytopoiesis. J. Exp. Med..

[3] Inra C.N., Zhou B.O., Acar M. (2015). A perisinusoidal niche for extramedullary haematopoiesis in the spleen. Nature.

[4] Lv H., Wang C., Liu Z. (2025). Suppression of the prostaglandin I2-type 1 interferon axis induces extramedullary hematopoiesis to promote cardiac repair after myocardial infarction. Circulation.

[5] Halade G.V., Norris P.C., Kain V. (2018). Splenic leukocytes define the resolution of inflammation in heart failure. Sci. Signal..

[6] Grosser T., Fries S., FitzGerald G.A. (2006). Biological basis for the cardiovascular consequences of COX-2 inhibition: Therapeutic challenges and opportunities. J. Clin. Invest..

[7] King K.R., Aguirre A.D., Ye Y.-X. (2017). IRF3 and type I interferons fuel a fatal response to myocardial infarction. Nat. Med..

[8] Dutta P., Courties G., Wei Y. (2012). Myocardial infarction accelerates atherosclerosis. Nature.

[9] Massa M., Rosti V., Ferrario,et M. (2005). Increased circulating hematopoietic and endothelial progenitor cells in the early phase of acute myocardial infarction. Blood.

[10] Emami H., Singh P., MacNabbet M. (2015). Splenic metabolic activity predicts risk of future cardiovascular events: demonstration of a cardiosplenic axis in humans. JACC Cardiov. Imag..

[11] Hulsmans M., Sager 1 H.B., Roh J.D. (2018). Cardiac macrophages promote diastolic dysfunction. J. Exp. Med..

[12] Robinette C.D., Fraumeni J.F. (1977). Splenectomy and subsequent mortality in veterans of the 1939-45 war. Lancet..

